# Nanostructurization and thermal properties of polyethylenes’ welds

**DOI:** 10.1186/s11671-015-0832-4

**Published:** 2015-03-19

**Authors:** Anatoliy Galchun, Nikolay Korab, Volodymyr Kondratenko, Valeriy Demchenko, Andriy Shadrin, Vitaliy Anistratenko, Maksym Iurzhenko

**Affiliations:** Plastics Welding Department, E.O.Paton Electric Welding Institute, National Academy of Sciences of Ukraine, B.8, 11, Bozhenko Str, 03680 Kyiv-150, Ukraine; Institute of Macromolecular Chemistry, National Academy of Sciences of Ukraine, 48 Kharkivske Avenue, 02160 Kiev, Ukraine

**Keywords:** Polyethylene, PE-63, PE-80, PE-100, Butt fusion, Welding, 81.20.Vj, 81.05.Lg, 81.07.-b

## Abstract

As it is known, polyethylene (PE) is one of the common materials in the modern world, and PE products take the major share on industrial and trade markets. For example, various types of technical PE like PE-63, PE-80, and PE-100 have wide industrial applications, i.e., in construction, for pipeline systems etc. A rapid development of plastics industry outstrips detailed investigation of welding processes and welds’ formation mechanism, so they remain unexplored. There is still no final answer to the question how weld’s microstructure forms. Such conditions limit our way to the understanding of the problem and, respectively, prevent scientific approaches to the welding of more complicated (from chemical point of view) types of polymers than PE. Taking into account state-of-the-art, the article presents results of complex studies of PE weld, its structure, thermophysical and operational characteristics, analysis of these results, and basing on that some hypotheses of welded joint and weld structure formation. It is shown that welding of dissimilar types of polyethylene, like PE-80 and PE-100, leads to the formation of better-ordered crystallites, restructuring the crystalline phase, and amorphous areas with internal stresses in the welding zone.

## Background

Construction of technological pipelines is one of the main fields of polymeric material application in the world [1]. Among polymers used for pipe production, polyethylene (PE) is one of the most commonly used [2]; this material has a perfect correlation between prices, mechanical properties, and weldability and, hence, has considerable advantage comparing with other polymers.

Pipes produced of various types of high-density polyethylene (HDPE; so-called “pipe” polyethylene) are used for pipeline construction [3]. Pipes for the first technological pipelines have been produced from the raw material marked as PE-63. Later on, the next brands, namely PE-80 and PE-100, have been developed and widely applied [4,5]. Currently, all these three types of polyethylene are used in the pipe industry [6].

Welding is the main method of PE pipes joining for pipeline construction. As for today, the following welding methods are sufficiently developed from technological point of view and are commonly used in practice: hot tool butt welding, hot tool socket welding, and resistance welding [7,8]. The last two methods require some special coupling details, such as socket and resistance fittings. Butt welding is the most simple and multipurpose method and can be used for pipes of all diameters (except the thin-wall pipes).

Performance characteristics of polyethylene pipelines are considerably dependent on the welded joint quality. As a rule, the declared pipeline lifetime is at least 50 years, and all factors that could promote pipe or weld destruction are permanently investigated and can be eliminated [9]. In cases when destruction has occurred, it is important to have an efficient and reliable repair technology [10]. Since pipes are produced of various types of polyethylene, it is required to develop welding technology providing reliable welds of dissimilar PE types.

All abovementioned welding methods have their own technological peculiarities and typical defects of welded joints [11]. Numerous scientific studies aim to improve hot tool butt welding method. Empiric methods are used by researchers in order to optimize main welding parameters for various technological conditions [12,13] as well as to investigate peculiarities of various-sized pipe welding [14]. Mechanical and thermal properties of the pipe material also strongly affect the hot tool butt welding process [15,16]. This should be taken into account when dissimilar types of polyethylene are welded with each other. PE-63, PE-80, and PE-100 have different technological characteristics, like, for example, shrinkage degree at cooling [17] and different melt flow indexes, so special welding technology and equipment should be developed for the cases when dissimilar PE types have to be welded together.

In spite of the numerous developed technologies and wide pipes’ welding practical application, the detailed research of polyolefin welding nature is still not completed; mechanism of welds’ formation is not explored sufficiently. Investigations of morphology, as a rule, enable to study the PE pipe macrostructure, fusion lines, and heat-affected zone geometry [18,19]. In some works, the PE macromolecular structure affecting on material weldability has been investigated [20] as well as the internal deformations in PE welded joints [21], but general mechanism of welded joint formation and macromolecular structures [22,23] in the weld are still studied insufficiently.

Hereby, there is still no complete understanding of PE and other polyolefin welded joint formation and structural peculiarities. Welding process of more complicated chemical system than polyethylene is even less explored. In this work, the results of complex investigations (by means of differential scanning calorimetry, thermogravimetric and thermomechanical analyses, as well as wide-angle X-ray scattering) of dissimilar technical PE type weld structure and their properties are presented. Basing on analysis of the results obtained, some new hypothesis concerning nature and mechanism of welds’ formation and polymer structuring in such welds are proposed.

## Methods

### Materials and processing

The following samples have been used for welding experiments, for structure analysis, and for investigations of mechanical and thermal properties: polyethylene pipes produced from two types of high-density polyethylene (HDPE) with different minimum required strengths (MRS)—PE-80 (MW_bimodal_ 300000 g/mol, density 0.953 g/cm^3^, MRS = 8 MPa), and PE-100 (MW_bimodal_ 300000 g/mol and density 0.960 g/cm^3^, MRS = 10 MPa).

**Figure 1:**
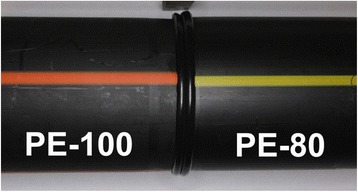
**The welded joint of PE pipes.** Dissimilar pipe weld (PE80 and PE-100, 63 mm in diameter).

The welding experiments have been carried out with 63 mm diameter and 6 mm wall thickness of PE-80 and PE-100 pipes using traditional hot plate butt welding under the following conditions: 200°C welding temperature, 0.2 MPa welding pressure, and 60 s upset time. Change over time was 3 s. The cooling time under pressure was 6 min. SAT-1 hot plate butt welding device produced by Experimental Welding Equipment Factory of E.O.Paton Electric Welding Institute of the NAS of Ukraine has been used for welding. Photo of PE-80 and PE-100 pipes’ weld is presented in Figure 1.

### Equipment and measurements

PE structure of PE (types PE-80 and PE-100) as well as of PE-80/PE-100 welds has been studied by means of wide-angle X-ray scattering (WAXS) using X-ray diffractometer DRON-4.07 (Burevestnik, Saint Petersburg, Russia) with X-ray optical scheme according to the Debye-Scherer method, using CuK_α_ emission (*λ* = 0.154 nm), monochromated by Ni-filter. X-ray tube BSV27Cu working at *U* = 30 kV and *I* = 30 mA has been used like a source of characteristic X-ray radiation. X-ray measurements have been carried out by step-by-step scanning with scattering angles (2*θ*) from 2.6° to 40°, with an exposure time of 5 s at temperature *T* = 20 ± 2°С.

Thermal properties of the initial specimens and of the welds have been explored by means of differential scanning calorimetry (DSC) on DSC Q2000 device from TA Instruments (New Castle, DE, USA), in the inert atmosphere (higher-purity nitrogen, GOST 9293–74) under temperatures from 40 to 200°C with linear heating rate 20°C/min. Specimen weight was between of 6 and 10 mg each. Temperature measurement precision was ±0.01°С, heat flow precision ±0.01 J/g.

Thermal stability and thermal-oxidative breakdown (TGA) of the initial specimens and of the weld have been studied with TGA Q50 device by TA Instruments (New Castle, DE, USA), in the dried air atmosphere under temperatures from 30 to 700°C with a linear heating rate of 20°C/min. Specimens’ weight was approximately 6–12 mg each. Temperature measurement precision was ±0.01°С, and weight loss precision was ±0.0001 mg.

Thermomechanical behavior and deformational characteristics (TMA) of the initial specimens and of the weld have been investigated with TMA Q400 EM device by TA Instruments (New Castle, DE, USA), in the dried air atmosphere, with a linear heating rate of 10°C/min under temperatures from 30 to 250°C. Measurements have been carried out in thermal expansion mode with the use of quartz indenter of 2.8 ± 0.01 mm in diameter. The applied indenter pressure to the specimen was permanent and equal to 10^−1^ MPa. Temperature measurement precision was ±0.01°С, and deformation control precision was ±0.01 μm. All the devices from TA Instruments have been certified according to the international standard ISO 9001:2000.

Mechanical properties (strength and elongation at break) of initial and welded specimens have been evaluated by means of tensile axial test (according to DBN B.2.5-41 standard) with a 50 mm/min tension rate at room temperature with FP-10 tension machine (Germany). Welding quality was also estimated basing on visual geometrical parameters. All investigations were repeated three times with different specimens for each time to enhance accuracy of the measurements.

## Results and discussion

**Figure 2:**
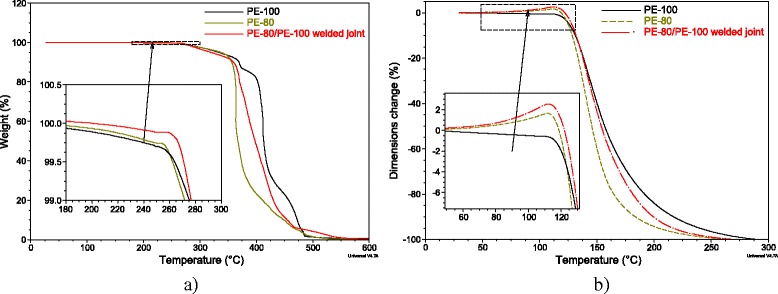
**Resulting plots of the TGA and TMA studies.** Thermogravimetric **(a)** and thermomechanical **(b)** results for pure PE-80, PE-100, and their PE-80/PE-100 weld.

Results of thermogravimetric investigations for PE-100, PE-80, and their weld are presented in Figure 2a. It is evidently that under temperatures 280-500°C, a curve of PE-80/PE-100 weld is located between curves of pure PE-80 and PE-100, which correspond to the thermal-oxidative breakdown process. Such behavior of curves is logical and is not a subject to any discussion. But in the starting area of thermal-oxidative breakdown process (up to 280°C), there is a certain increased stability of PE-80/PE-100 weld comparing to the pure polyethylenes. As one can see in the insert in Figure 2a, PE-80/PE-100 weld has lower weight loss in the beginning of the breakdown and increased (up to 10°C) temperature of the breakdown start comparing both with PE-80 and PE-100. Such curve’s pattern indicates that some structures with higher thermal stability are formed in the weld.

A similar behavior of materials is observed in the thermomechanical test (Figure 2b). Relative strain curve of PE-80/PE-100 weld during the melting at temperatures higher than *T* = 140°C is located between respective curves for pure PE-80 and PE-100. Wherein under temperatures 25-130°C, PE-80/PE-100 weld has the maximum values of thermal expansion (insert in Figure 2b) comparing to the pure PE-80 and PE-100. It can be explained by the existence of the internal stresses in “frozen” areas of the amorphous part of polymer, which appear during welding process. Relaxation and unfreezing of these areas at heating lead to enhancing of molecular mobility and increasing of the material volume.

**Figure 3:**
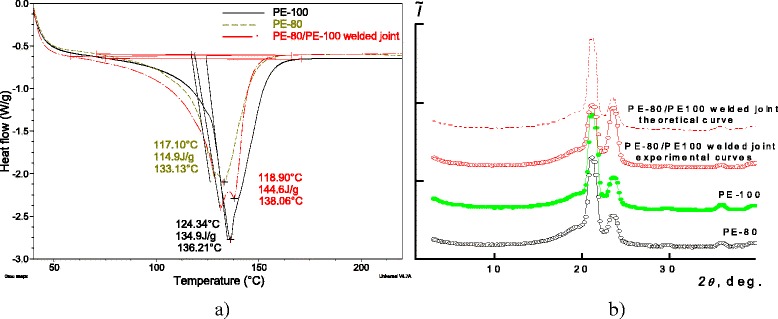
**Resulting plots of the DSC and WAXS studies.** DSC **(a)** and WAXS **(b)** spectra of pure PE-80, PE-100, and of their PE-80/PE-100 weld.

Basing on the abovementioned data, we can assume that under welding of dissimilar types of polyethylene, like PE-80 and PE-100, areas with higher thermal stability (evidently, in crystalline phase) and areas with internal stresses (in amorphous phase) are formed in the welding zone. In order to verify this idea, all specimens (both pure polyethylene types and their welds) have been studied by means of differential scanning calorimetry (Figure 3a) and wide-angle X-ray spectroscopy (Figure 3b).

For all three specimens on DSC curves, two minimums corresponding to the melting processes of crystalline structures within PE can be observed, wherein these two melting temperatures at all specimens indicate their polycrystallinity. The first minimum *T*_*m*1_ indicates the melting point for the easier-melting fraction with a melting temperature of 117-125°C. The second minimum *T*_*m*2_ corresponds to the melting of better-ordered (better-packed) crystallites with higher thermal stability with melting temperature between 133°C and 138°C. Melting temperatures corresponding to both types of crystallites for all specimens are presented in Table 1.

**Table 1:** **Thermal characteristics (melting temperatures and enthalpies) of both types of polyethylenes and of their weld obtained from DSC studies**

**Specimen**	**Melting temperature** ***Т***_***m*1**_ **, °С**	**Melting temperature** ***Т***_***m*2**_ **, °С**	**Melting enthalpy, J/g**
PE-80	117.10	133.13	114.90
PE-100	124.34	136.21	134.90
PE-80/PE-100 weld	118.90	138.06	144.60

Increased melting temperature *T*_*m*2_ of the weld comparing to respective *T*_*m*2_ of both pure polyethylene types is an important confirmation of assumption that the weld contains areas with higher thermal stability and, respectively, with crystallites with higher order (packing).

Similar trend is also found for integral melting enthalpies, defined from the melting areas on DSC curves that enabled us to calculate degree of crystallinity using the classical equation [24] (see Table 1). For PE-80/PE-100 weld integral melting enthalpy is the biggest among three polymers that evidently indicates higher thermal stability of crystalline phase of the PE-80/PE-100 weld comparing both with pure PE-80 and PE-100, and, in turn, this can be explained by formation of better-packed crystallites in the welded joint. Degrees of crystallinity presented in Table 2 are calculated basing on the integral melting enthalpies for each specimen using classical equation [24]. One can see that the degree of crystallinity of the weld is the highest among three specimens and, respectively, higher than of pure matrixes of both PE types.

**Table 2:** **Structural and mechanical characteristics of polyethylenes and of their weld**

**Specimen**	**Degree of crystallinity (DSC) %**	**Degree of crystallinity (WAXS) %**	**Crystallites size** ***L*** **1 (2** ***θ*** **= 21.2°) nm**	**Crystallites size** ***L*** **2 (2** ***θ*** **= 23.6°) nm**	**Tensile strength MPa**	**Relative tensile strength %**
PE-80	42	56	7.2	7.2	19.6	100
PE-100	51	57	7.2	7.2	23.1	100
PE-80/PE-100 weld	53	66	7.2	8.0	Destroyed on the basic material	>100

Other arguments, which confirm our assumption, are WAXS results (Figure 3b). PE-80, PE-100, and PE-80/PE-100 weld’s spectra analysis show that they have amorphous-crystalline structure (presented by diffraction maximums at scattering angles 2*θ*max = 21.2°, 23.6°, 29.7°, and 36.7° against the background of virtual amorphous halo).

Relative crystallinity degree (*X*cr) was determinated by Matthews’s method [25]:

1 $$ {X}_{\mathrm{cr}}={Q}_{\mathrm{cr}}{\left({Q}_{\mathrm{cr}}+{Q}_{\mathrm{am}}\right)}^{-1}\cdot 100 $$

where *Q*cr is the area of diffraction maximums describing crystalline structure of polymer and *Q*_cr_ + *Q*_am_ is the total area of diffraction pattern within the scattering angles, where amorphous-crystalline structure of polymer is appearing. This determination has shown that crystallinity degree for both PE-80 and PE-100 is almost equal (approximately 56% for PE-80 and 57% for PE-100) and is quite different to such degree of the PE-80/PE-100 weld (66%), and these data correlate with results of DSC investigations. Differences in crystallinity degrees calculated from DSC and WAXS studies, as reported in [26], are quite typical and can be explained by unequal research conditions and macromolecule state at room (WAXS) and elevated (DSC) temperatures.

In turn, effective crystallite size (*L*1 and *L*2) evaluation, made by Scherer’s method [27], is presented as follows:

2 $$ L=K\lambda {\left(\beta \cos {\theta}_{\max}\right)}^{-1} $$

where *K* is a constant related to the crystallite’s shape (if shape is not determined, *К* = 0.9), and *β*, which is the angular half-width (width of half-height) of diffraction maximum, has shown that average values of *L*1 ≈ 7.2 nm for PE-80, PE-100, and PE-80/PE-100 weld and average values of *L*2 ≈ 7.2 nm for PE-80 and PE-100, while for PE-80/PE-100 weld, *L*2 ≈ 8.0 nm (diffraction maximums at 2*θ*max = 21.2° and 23.6° have been used for the calculation).

In order to evaluate the difference between the experimental X-ray diffraction pattern of PE-80/PE-100 weld and diffraction patterns of PE-80 and PE-100 mechanical blends (under conditions of zero interaction between them), further calculations of such blends’ X-ray diffraction pattern have been done assuming that both components (both types of PE) are making an additive contribution into the diffraction picture:

3 $$ {I}_{\mathrm{add}}={w}_1{I}_1+{w}_2{I}_2 $$

where *I*_1_ and *I*_2_ are intensities of wide-angle X-ray scattering of PE-80 and PE-100; *w*_1_ and *w*_2_ are mass content of the components in the system (*w*_1_ + *w*_2_ = 1). Comparing experimental and calculated X-ray diffraction patterns, one can see in Figure 3 that non-additive change in the experimental diffraction curve comparing with the theoretical one takes place; it is an important result since it confirms that interaction between PE-80 and PE-100 macromolecules occurs in the PE-80/PE-100 weld. Analyzing PE-80/PE-100 weld experimental diffraction curve, it is obvious that intensity of the first diffraction maximum (2*θ*max = 21.2°) is decreasing and intensity of the second diffraction maximum is considerably increasing (2*θ*max = 23.6°) comparing to the respective diffraction maximums on both pure PE spectrums. Apparently, this factor indicates that the restructuring of PE-80 and PE-100 crystalline phases occurs when these two materials are welded and that better-packed crystallites (comparing to the pure materials) are formed in PE-80/PE-100 weld. By this fact, the increased strength of dissimilar polymers joint identified earlier by specialists and confirmed experimentally before the start of current investigations can be explained (see Table 2). The values of crystallite size (*L*1 and *L*2) for each specimen calculated basing on separate diffraction maximums are also presented in Table 2. Thus, the increased crystallite size characterizes for PE-80/PE-100 weld.

## Conclusions

Results of complex thermal and structural investigations for two technical PE types (PE-80 and PE-100) and of their weld have been represented. The welded joint was produced by means of traditional hot tool butt welding. It is revealed that during the welding process, restructuring of crystalline phases occurs and crystalline areas with higher mechanical and thermal properties appear due to the increase of quantity of crystallites and to their bigger size and better ordering (packing).

## Abbreviations

DSC: differential scanning calorimetry
HDPE: high-density polyethylene
PE-80: high-density polyethylene with MW 80000 g/mol and density 0.953 g/cm^3^
PE-100: high-density polyethylene with MW 100000 g/mol and density 0.960 g/cm^3^
PE: polyethylene
TGA: thermogravimetric analysis
TMA: thermomechanical analysis
WAXS: wide-angle X-ray scattering
